# Multi-Illumination Single-Holographic-Exposure Lensless Fresnel (MISHELF) Microscopy: Principles and Biomedical Applications

**DOI:** 10.3390/s23031472

**Published:** 2023-01-28

**Authors:** José Ángel Picazo-Bueno, Martín Sanz, Luis Granero, Javier García, Vicente Micó

**Affiliations:** 1 Department of Optics, Optometry and Vision Science, University of Valencia, 46100 Burjassot, Spain; 2 Biomedical Technology Center of the Medical Faculty, University of Muenster, Mendelstr. 17, D-48149 Muenster, Germany

**Keywords:** multiplexed holography, digital holography, in-line holographic microscopy, Gabor holography, lensless microscopy, digital image processing, label-free imaging, biomedical imaging

## Abstract

Lensless holographic microscopy (LHM) comes out as a promising label-free technique since it supplies high-quality imaging and adaptive magnification in a lens-free, compact and cost-effective way. Compact sizes and reduced prices of LHMs make them a perfect instrument for point-of-care diagnosis and increase their usability in limited-resource laboratories, remote areas, and poor countries. LHM can provide excellent intensity and phase imaging when the twin image is removed. In that sense, multi-illumination single-holographic-exposure lensless Fresnel (MISHELF) microscopy appears as a single-shot and phase-retrieved imaging technique employing multiple illumination/detection channels and a fast-iterative phase-retrieval algorithm. In this contribution, we review MISHELF microscopy through the description of the principles, the analysis of the performance, the presentation of the microscope prototypes and the inclusion of the main biomedical applications reported so far.

## 1. Introduction

Light microscopy has been used for centuries to provide numerous discoveries in biomedicine at the micro- and nano-scale. Therein, optical compound microscopes are typically the main tool for obtaining high-quality imaging in real time. However, their performance normally relies upon fairly bulky, complicated and expensive lenses and well-aligned opto-mechanical parts, which restrict their applicability in field settings and laboratories with low resources [1]. Hence, new low-cost, field-portable and easy-to-use medical devices are of particular interest in global healthcare and point-of-care diagnosis in order to provide early and accurate diagnosis [2]. In that regard, lensless holographic microscopes are ideal candidates since they digitally reconstruct images of microscopic specimens without using any lenses, so that their designs are compact, lightweight and inexpensive [3,4,5]. New findings in economical optoelectronic components have recently pushed towards a strong development of lensless holographic microscopes [6,7,8,9,10,11,12,13,14,15,16,17,18,19,20,21,22] and a huge variety of biological applications, including cancer, SARS-CoV-2 and disease diagnosis [23,24,25], water and air quality monitoring [7,15,26,27], microbial viability testing [21,28], or 3D motion tracking of biological samples [20,29,30,31,32,33], among others [9,10,11,17,34].

Lensless holographic microscopy (LHM) comes from the digitalization of Gabor’s concept of holography reported about 70 years ago [35]. LHM proposes an extremely simple optical layout, where only a coherent point illumination source and a digital camera are utilized to illuminate the specimen and to record the resulting diffracted wavefront, respectively [36]. Under certain approximations [37,38], holographic (intensity and phase) image reconstruction is achieved from the recorded Fresnel diffraction pattern (in-line digital hologram) by means of computational methods [39]. Holographic principles of LHM enable digital refocusing within a sample volume and thus three-dimensional (3D) recovery at extended depth-of-field (DOF). 

Inside the extreme simplicity of LHM configurations, there exists great flexibility in the choice of both point light sources [6,7,22,34,36,40,41,42,43,44,45] and layout geometries [6,7,15,16,19,20,34,40,42,43,44,46]. Concerning illumination, point sources have been generated in LHM mainly using pinholes in combination with laser beams [7,40,41,47] or LEDs [15,34], high-numerical-aperture (NA) focusing lenses together with either LEDs [6] or laser diodes [14,22,48], and also employing different types of lenses and light sources such as GRIN lenses [21,42], tunable lenses [36], optical fibers [43,49], pulsed lasers [44], SLED sources [45] and terahertz lasers [50]. Regarding layout geometry, LHM has been typically implemented under two opposite configurations [3]. One of them is normally implemented in digital lensless holographic microscopy (DLHM) and sets the specimen much nearer to the point illumination than to the digital camera [6,7,40,42,44,47]. Thus, the sample diffraction wavefront is magnified by geometric projection and the numerically reconstructed images are similar in magnification (*M*~5-20X) and resolution to those achieved by digital holographic microscopy when considering objective lenses of *NA*~0.3–0.5 [51], although higher resolution images have been also reported [52,53,54]. In the opposite layout geometry, employed in lensless on-chip microscopy, the object is placed very close to the digital camera and relatively far from the illumination point source [15,16,19,20,34,43,46]. In this case, the magnification approaches 1X and the recorded field of view (FOV) is therefore enormous and equal to the entire digital sensor area; however, although several pixel super-resolution approaches have been proposed [43,55,56], the resolution is usually modest (*NA*~0.2) and directly depends on the digital camera pixel size. Those two opposite geometries are the most common LHM arrangements, but intermediate cases have been also reported [9,57].

As an in-line holographic microscopy technique, LHM is negatively affected by coherent noise, presumption of weak diffraction and the presence of the twin image: factors that reduce the quality of the image reconstructions [38]. On one hand, coherent noise is inherent to the usage of coherent illuminations and is composed of a combination of speckle noise, coherent artifacts and multiple reflection interferences; however, it can be reduced by either overlapping several in-line holograms [58,59] or using partially coherent sources [6,45,60,61]. On the other hand, weak diffraction assumption forces the sample to be sparse and almost transparent and can even prevent imaging, significantly decreasing the variety of samples to be imaged by LHM; however, this assumption can be avoided by either implementing specific phase-shifting holographic techniques [38,62], employing multi-height phase-retrieval procedures [63,64,65,66,67,68,69,70,71,72,73] or reinserting an external reference wave as in an off-axis configuration [74,75,76]. Finally, twin image presence is inherent to in-line holographic configurations [77], and its defocused version spatially overlaps with the reconstructed image, thus disturbing the final image quality. Twin image presence can be removed in LHM by following the same phase-shifting [38,62] or iterative multi-height phase-retrieval [63,64,65,66,67,68,69,70,71,72,73] strategies applied for avoiding weak diffraction assumption. However, in those approaches, several holograms must be sequentially recorded by either mechanically changing the sample-to-sensor distance [43,63,64,73,78], or scanning the illumination source aperture [79], or changing the illumination wavelength [65,67,80,81,82] or changing the illumination angle [67,83] or inducing image defocus using a SLM [66], thus preventing the analysis of fast, dynamic events. 

In addition to those sequential phase-retrieval methods, there exist others that remove the presence of the twin image from a single capture [34,72,84]. For instance, Waller et al. proposed a method based on the transport of intensity equation (TIE), in which the three images with slight defocus required for TIE phase retrieval were simultaneously achieved by using broadband illumination, imaging lenses with chromatic aberrations and a common red-green-blue (RGB) camera [72]. Also in 2010, Mudanyali et al. employed a phase-retrieval method based on object mask constraints, where a two-dimensional (2D) mask on the object plane was defined by thresholding, and the twin-image was iteratively removed [34]. The 2D object mask was also defined in another approach by recording an auxiliary image of the sample employing a design combining a conventional bright-field microscope and LHM [85]. Later on, Noom et al. reported on a multi-illumination iterative phase-retrieval method based on wavelength dependence of diffraction, where three in-line Fresnel holograms were simultaneous achieved employing a RGB fiber-coupled source containing three diode lasers and a color digital sensor [84]. More recently, several off-axis lensless approaches have appeared to solve the twin-image problem, where the phase is recovered from an off-axis Fresnel hologram by conventional Fourier filtering and further backpropagation [74,75,76]. Finally, there are also emerging single-shot phase-retrieval methods based on deep-learning and convolutional neural networks that have achieved excellent twin-image removal after some training [86]. 

In line with those single-shot phase-retrieval methods applied to LHM, the technique called multi-illumination single-holographic-exposure lensless Fresnel (MISHELF) microscopy proposes a new concept of LHM based on wavelength multiplexing and the use of a rapid-convergence iterative phase-retrieval algorithm for coherent noise reduction and twin-image mitigation [22,47,48,49]. MISHELF microscopy illuminates the sample with various wavelengths and records the different in-line Fresnel holograms in a single camera acquisition. So far, MISHELF microscopy has been reported using two [48], three [22,47] and four [49] illumination/detection channels, validated either at the laboratory level on an optical table within a well-controlled environment [47,48], or at the field level in different 3D-printed prototype microscopes [22,49], as well as applied in biomedical research for sperm motility assessment [33].

In this manuscript, we aim to provide an overview of MISHELF microscopy. We first describe the basic principles of the technique regarding the hardware and software (Section 2). Considering the hardware, we describe the optical layout and the most important optical parameters to be considered when implementing MISHELF microscopy (Section 2.1). Regarding the software, we provide a complete and generalized description of the recording process (Section 2.2) and the iterative algorithm for complex amplitude reconstruction (Section 2.3). In Section 3, a performance analysis of the technique is included (Section 3.1) as well as a comparison of the performance when MISHELF microscopy is implemented using different numbers of illumination/detection channels (Section 3.2). In addition, we provide a brief overview of the microscope prototypes designed in Section 4. Furthermore, in Section 5, the biomedical applications of MISHELF to sperm motility analysis have been presented. Finally, Section 6 includes a summary of the technique, indicating some positive and negative aspects and concluding with an outlook of MISHELF microscopy.

## 2. Description of MISHELF Microscopy

### 2.1. Optical Layout

**Figure 1:**
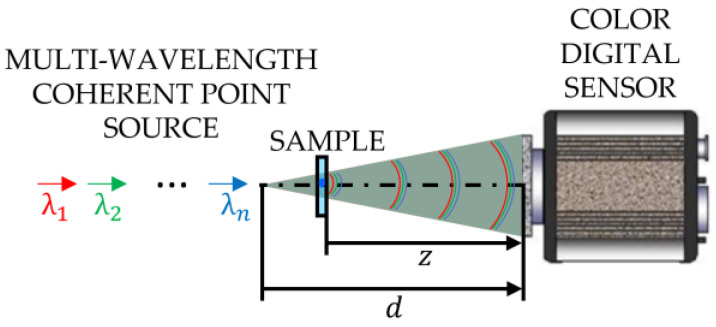
Optical scheme implemented in MISHELF microscopy.

MISHELF microscopy implements a typical LHM layout based on a DLHM configuration, in which the sample under analysis is placed much nearer to the illumination point than to the digital camera. The optical scheme of MISHELF microscopy is depicted in Figure 1, where a multi-wavelength coherent point light source illuminates a weakly diffractive sample with different wavelengths $\lambda_{i=1,2,\ldots ,n}$ in transmission. For each illumination, the light diffracted by the specimen interferes with the non-diffracted light to produce an in-line interference pattern. Then, the geometrically magnified versions of such interferograms are simultaneously captured by a color digital camera, thus providing a color Gabor digital hologram. 

In MISHELF microscopy, several ways to produce a multi-wavelength coherent point source have been reported [22,47,48,49]. First, the light coming from different lasers with different wavelengths has been combined before arriving at a pinhole with dichroic beam combiners [47]. Second, single multi-illumination devices, either with fiber-coupled [48] or with can-type [22] formats, containing different laser diodes have been employed together with a high-NA focusing lens. Finally, a multi-wavelength source containing different laser diodes coupled to a single mode fiber has been used without any additional lens or pinhole [49]. It is worth noting that high-NA focusing lenses or pinholes with small apertures are usually employed in DLHM to increase the NA of the point source [41,87]. Regarding the color digital sensor, board-level cameras (CCD or CMOS sensor type) with a Bayer mosaic filter mask have been employed for the recording of two- or three-color in-line holograms [22,47,48], whereas four-color holograms have been recorded with a camera containing a Kodak Truesense red-green-blue-white (RGBW) sensor [49].

In the extremely simple layout implemented in MISHELF microscopy, the fundamental optical parameters depend on the different geometrical and/or digital features. Hence, magnification (M), field of view (FOV), numerical aperture (NA) and spatial resolution (ρ) are directly defined by the axial distances between the different elements (sample–digital sensor, (z); and point source–digital sensor, (d)) and by the physical dimensions of the digital sensor (pixel pitch, (p); and number of pixels, $(N_{x},\;N_{y}$)). As in DLHM, the magnification M is defined by the geometrical projection of the object onto the sensor plane, so that M is determined by the distances d and z through the expression

(1) $$M=\frac{d}{d-z}$$

The axial distances d and d−z are typically set to 10–20 mm and 0.5–2 mm, respectively, and, therefore, the M values usually range from 10 X to 20 X in MISHELF microscopy. However, MISHELF microscopy is not limited to those values and either higher or smaller M can be easily achieved by simply modifying such distances.

Furthermore, the FOV is determined by M and the whole digital sensor area ($N_{x}p\times N_{y}p$), and can be calculated as

(2) $$FOV=\frac{N_{x}N_{y}p^{2}}{M^{2}}$$

Based on the previously mentioned M values, the expected FOV is usually similar to that achieved by using conventional microscopy with 10–20 X microscope lenses, and usually takes around 300 × 300–600 × 600 μm^2^ when considering conventional digital sensors (p~2–6 μm, and $N_{x,y}$~1000–2000 pixels).

In addition, the NA of the optical system depends on z and half of the longitudinal size of the sensor area ($N_{x,y}p/2$) as

(3) $$NA=\sin\left[\tan^{-1}\left(\frac{N_{x,y}p}{2z}\right)\right]$$

The NA becomes higher when either the sample approaches the sensor (reducing z) or the sensor becomes bigger (increasing $N_{x,y}p/2$) and usually takes values around NA~0.2–0.3. 

Meanwhile, ρ depends on several factors such as diffraction, digital sampling, limited illumination coherence and signal-to-noise ratio (SNR) [88]. Considering that MISHELF microscopy introduces enough M to satisfy the Nyquist–Shannon sampling theorem [89], employs high spatial and temporal coherence light sources, and provides high SNR images, ρ is only limited by diffraction and is given by the expression

(4) $$\rho =k\frac{\lambda }{NA}$$

k being a constant parameter. In the case of MISHELF microscopy, this parameter can be regarded as k~1, since this is the value considered in near-field imaging under coherent illumination following the Rayleigh criterion [90]. Considering the previous NA values, typical MISHELF resolution limits for usual red (630 nm), green (520 nm) and blue (450 nm) illuminations are $\rho_{R}\sim 3.15-2.10\;\mu m$, $\rho_{G}\sim 2.60-1.73\;\mu m$ and $\rho_{B}\sim 2.25-1.50\;\mu m$, respectively. Although the different illumination wavelengths required for MISHELF implementation provide different resolution limits, MISHELF microscopy is not penalized in that aspect since its spatial resolution is determined by the resolution provided by the shortest wavelength (typically blue 450 nm or violet 405 nm illuminations), as we will see in Section 3.1.

Hence, considering all those parameters and an illumination with a high enough NA to illuminate the entire sensor area, the best scenario for MISHELF microscopy, from an imaging point of view, is that both ρ and FOV are optimized with respect to each other. On one hand, ρ optimization is achieved when using short λ (blue or violet) for illumination, and short sample–sensor distance z and big sensor areas with a high $N_{x,y}p$ product for defining a high NA. On the other hand, to increase the FOV, we must consider low V (low ratio between d and d−z distances) and a high $N_{x,y}p$ product. However, to avoid the resolution limit defined by digital sampling, ρ>2p/M, and M must not be below a certain value imposed by the pixel pitch p. Hence, the optical layout of MISHELF microscopy is optimized when using short illumination wavelengths λ, short sample-to-sensor distances z, big sensor areas $N_{x,y}p$, small pixel pitches p, and optimum point source-to-sample distances d−z to optimize both ρ and FOV at the same time. It is also worth pointing out that MISHELF microscopy also requires color digital sensors which normally include Bayer color filter arrays, so that digital sampling will be more restrictive for blue and red illuminations than for green ones.

### 2.2. Recording of Color In-Line Gabor Holograms

The optical setup defined in MISHELF microscopy is aimed to provide multi-wavelength in-line Gabor holograms recorded with a color digital camera. Thus, the intensity distribution of each hologram is given by

(5) $$I^{\lambda_{i}}\left(x,y\right)={\left|A_{ref}^{\lambda_{i}}\left(x,y\right)\right|}^{2}+{\left|A_{obj}^{\lambda_{i}}\left(x,y\right)\right|}^{2}+A_{ref}^{\lambda_{i}}\left(x,y\right)A_{obj}^{*,\;\lambda_{i}}\left(x,y\right)\;+A_{ref}^{*,\;\lambda_{i}}\left(x,y\right)A_{obj}^{\lambda_{i}}\left(x,y\right)$$

$A_{ref}^{\lambda_{i}}\left(x,y\right)$ and $A_{obj}^{\lambda_{i}}\left(x,y\right)$ being the complex amplitudes of the reference and object waves, respectively, for a particular wavelength $\lambda_{i}$ at the recording plane (considered as plane z=0) with (x,y) spatial coordinates, and * the conjugate of the complex amplitude.

On the right side of Equation (5), the first term represents the intensity distribution coming from the reference wave. This wave has a spherical shape given by the illumination point and covers the whole sensor area, so that it produces a radial reduction of the background intensity, which can be easily compensated/subtracted with the recording of an image without an object. The second term corresponds to the light intensity diffracted by the sample. The weak diffraction assumption imposed on the sample in LHM, and therefore in MISHELF microscopy, forces to the sample to be sparse and semi-transparent for each and every wavelength, meaning that $A_{ref}^{\lambda_{i}}\left(x,y\right)\gg A_{obj}^{\lambda_{i}}\left(x,y\right)$ for all illuminations employed. When this condition is satisfied, the light intensity diffracted by the sample is weak compared to the non-diffracted light, so that this second term in Equation (5) is small enough ${\left|A_{obj}^{\lambda_{i}}\left(x,y\right)\right|}^{2}\ll A_{ref}^{*,\;\lambda_{i}}\left(x,y\right)A_{obj}^{\lambda_{i}}\left(x,y\right)$ and can be neglected. Finally, the third and fourth terms represent the interferometric terms and possess information about the twin (real and virtual) images of the sample. Since they are complex conjugates of each other, those twin images appear located at the same distance but symmetrically from the hologram plane, so that the recovery of the complex amplitude (amplitude and phase) of one of those images through a digital back-propagation will be altered by the defocus version of the other twin image. This twin image presence can be considered as an undesired artifact, and its removal (or minimization) improves the quality and accuracy of the complex amplitude reconstructions in LHM. In MISHELF microscopy, the mitigation of the twin image is carried out by the implementation of the following iterative algorithm.

### 2.3. Digital Image Processing

**Figure 2:**
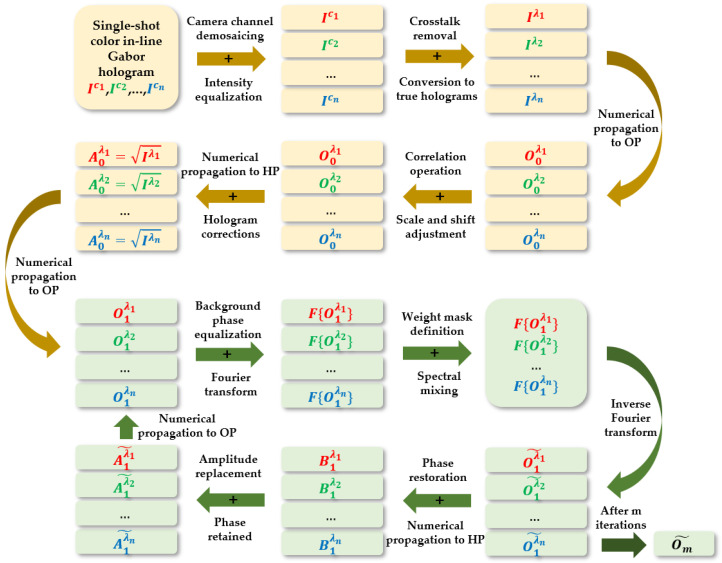
Flow chart of the algorithm implemented by MISHELF microscopy. In the chart, the variables I, A and B mean intensity and complex amplitudes at the hologram plane (HP). O represents the complex amplitude at the object plane (OP) and F{} is the Fourier transform operator. All variables are (*x*, *y*) spatially dependent.

In MISHELF microscopy, complex amplitude retrieval with minimized twin image presence is achieved with a rapid-convergence iterative algorithm. The algorithm workflow diagram is depicted through Figure 2. Note that in Figure 2, all variables are dependent on the (x,y) coordinates, but they are not included to save space. The algorithm can be split into two main parts (top yellow and bottom green parts in Figure 2). The former block performs a digital preparation of the color holograms before proceeding to the latter part, which carries out complex amplitude recovery by the application of an iterative algorithm. 

#### 2.3.1. Preprocessing Process

The first block starts once a color in-line Gabor hologram is recorded in a single camera snapshot with a polychromatic digital sensor, in which the same number of detection channels ($c_{1},\;c_{2},\;\ldots ,\;c_{n}$) as wavelengths ($\lambda_{1},\;\lambda_{2},\;\ldots ,\;\lambda_{n}$) are used for sample illumination. This illumination/detection matching is fundamental for the implementation of the algorithm. After that, knowing the pixel arrangement in the camera filter array, a demosaicing process is performed to separately extract the information coming from the different recording channels of the camera, thus obtaining n color-coded holograms to be processed separately ($I^{c_{1}},\;I^{c_{2}},\;\ldots ,\;I^{c_{n}}$). Once separated into the different channels, an intensity equalization stage is performed to each individual hologram in order to homogenize the illumination profiles throughout the frame (to correct the low intensity at the edges of the hologram). In essence, this stage consists of the subtraction of the first term of Equation (5) by the obtention of a single background image (without sample), which can be initially recorded in a preliminary calibration process.

After intensity homogenization, the crosstalk coming from the spectral response of the different camera channels must be subtracted to remove the contribution resulting from the other illumination wavelengths and, therefore, having a detuned illumination/detection scheme, which improves the image quality in the reconstruction process. For a better understanding, one can suppose that the crosstalks are not eliminated. Thus, each of the holograms will include the twin images provided by the illumination of interest at that channel as well as the twin images provided by the other spurious wavelengths. That will decrease the hologram dynamic range, so that the contrast is reduced. In addition, that will also introduce into the reconstructed image a significant fluctuation in the background caused by the out-of-focus images, resulting in a notable perturbation in the complex amplitude distribution. 

In general, each illumination contributes to each of the channels with distinct efficiency according to the spectral response. Hence, the true color holograms related to the real wavelengths can be achieved in two manners. On the one hand, we can consider the spectral sensitivity provided by the manufacturer to estimate the quantity of a certain wavelength detected by the distinct camera channels. This is an approximate approach to obtain the set of the n×n coefficients that relates the amount of each of the wavelengths entering into each of the detection channels. Thus, one can recover the true illumination holograms with a weighted subtraction operation considering the *n* × *n* coefficients. On the other hand, the recovery of the true illumination holograms can be likewise achieved by calibration. This second procedure is usually preferred because it is a more accurate approach to obtain the n×n coefficients rather than taking them from a plot. This calibration is performed before introducing the sample and setting the illumination intensities for the different light sources, and then the individual illuminations are sequentially switched on in order to capture n distinct images. After that, the set of n×n coefficients are calculated from the mean values of the intensity distributions for each of the channels.

In essence, the intensity distributions of the distinct detection channels ($I^{c_{1}},\;I^{c_{2}},\;\ldots ,\;I^{c_{n}}$) come from a combination of the *n* illumination intensities ($I^{\lambda_{1}},\;I^{\lambda_{2}},\;\ldots ,\;I^{\lambda_{n}}$) and can be written as a matrix:

(6) $$\left(\begin{array}{c} \begin{array}{c} I^{c_{1}}\\ I^{c_{2}} \end{array}\\ \cdots \\ I^{c_{n}} \end{array}\right)=\left(\begin{array}{ccc} \begin{array}{c} a_{1}\\ a_{2} \end{array} & \begin{array}{c} b_{1}\\ b_{2} \end{array} & \begin{array}{c} \begin{array}{cc} \cdots & l_{1} \end{array}\\ \begin{array}{cc} \cdots & l_{2} \end{array} \end{array}\\ \cdots & \cdots & \begin{array}{cc} \cdots \;\; & \cdots \end{array}\\ a_{n} & b_{n} & \begin{array}{cc} \cdots & l_{n} \end{array} \end{array}\right)\cdot \left(\begin{array}{c} \begin{array}{c} I^{\lambda_{1}}\\ I^{\lambda_{2}} \end{array}\\ \cdots \\ I^{\lambda_{n}} \end{array}\right)$$

or, equivalently,

(7) $$\vec{I^{C}}=L\cdot \vec{I^{\lambda }}$$

Note that L is a matrix whose factors represent the quantity of each light detected by each channel. The coefficients ($a_{i},\;b_{i},\;\ldots ,\;l_{i}$) depict the sensitivity of the i detection channel to the i illumination, being i=1, 2, …, n. Those coefficients need only be calculated once in a preliminary calibration procedure. After obtaining the detector response matrix (L), one can compute the true intensity distributions by considering the inverse matrix of L as follows:

(8) $$\vec{I^{\lambda }}=L^{-1}\cdot \vec{I^{C}}$$

The same process must be conducted on each one of the captured color holograms for proper crosstalk removal. After that, the true $I^{\lambda_{i}}$ holograms coming from the real illumination wavelengths are achieved.

It could also happen that the point light sources coming from the different wavelengths are not located at exactly the same spatial position, but they could be laterally or axially shifted in relation to one another. In that case, the images brought into focus ($O_{0}^{\lambda_{i}}$) by numerical backpropagation will present different magnifications and/or be laterally shifted in relation to one another. Therefore, rescaling and shifting calibration procedures should be performed on the focused images to compensate for this effect. Correlation operations can be used between all the focused images to adjust one another in terms of magnification and centering. Then, the scale and shift factors should be stored and applied, after numerical propagation, to the corresponding true holograms ($I^{\lambda_{i}}$) to remove the different magnifications and displacements. The resulting true holograms are achieved as though they come from a single point light source with different illumination wavelengths (as when a pinhole is used). Finally, the true holograms are employed as inputs into the MISHELF iterative algorithm (green second block at Figure 2).

#### 2.3.2. Iterative Algorithm for Complex Amplitude Retrieval

The iterative part begins by considering the square root of the intensity distribution of each true hologram to define the amplitudes per each wavelength ($A_{0}^{\lambda_{1}}$, $A_{0}^{\lambda_{2}}$, …, $A_{0}^{\lambda_{n}}$) as

(9) $$A_{0}^{\lambda_{i}}\left(x,y\right)=\sqrt{I^{\lambda_{i}}\left(x,y\right)}$$

Then, each individual amplitude is propagated towards its best focus plane by using one of the existing methods to numerically solve the diffraction integral [39,89], generating n object images. Among them, we can use, for instance, the diffraction Rayleigh–Sommerfeld integral employing a convolution operation [91] by using the following expression:

(10) $$O_{1}^{\lambda_{i}}\left(x_{o},y_{o};-z\right)=F^{-1}\left\{F\left\{A_{0}^{\lambda_{i}}\right\}\left[k_{x},k_{y}\right]\;\;\cdot \;F\left\{\frac{1}{2\pi }\frac{e^{jk_{i}r}}{r}\frac{\left(-z\right)}{r}\left(\frac{1}{r}-jk_{i}\right)\right\}\left[k_{x},k_{y}\right]\right\}\left[x_{o},y_{o}\right]$$

where F{} and $F^{-1}\{\}$ are the 2D Fourier and inverse Fourier transforms, respectively, ($k_{x},k_{y})$ are the spatial frequencies, $k_{i}$ is the wavenumber $k_{i}=2\pi /\lambda_{i}$, $r=\sqrt{x^{2}+y^{2}+z^{2}}$, and $\left(x_{o},y_{o}\right)$ are the spatial coordinates at the object plane. $O_{1}^{\lambda_{i}}\left(x_{o},y_{o};-z\right)$ is a complex distribution which includes, in addition to other spurious contributions, the complex amplitude at the object plane. From $O_{1}^{\lambda_{i}}\left(x_{o},y_{o};-z\right)$, the amplitude distribution is computed as its modulus $\left|O_{1}^{\lambda_{i}}\left(x_{o},y_{o};-z\right)\right|$ and the phase distribution can be computed as

(11) $$\varphi_{1}^{\lambda_{i}}\left(x_{o},y_{o};-z\right)=\tan^{-1}\left\{\frac{Im\left\{O_{1}^{\lambda_{i}}\left(x_{o},y_{o};-z\right)\right\}}{Re\left\{O_{1}^{\lambda_{i}}\left(x_{o},y_{o};-z\right)\right\}}\right\}$$

In Equation (11), $\tan^{-1}\{\}$, Im{} and Re{} are the arctangent function, and the imaginary and real parts of a complex function, respectively.

In addition, the resultant propagation distances must be stored. The wavelength dependence of refraction makes the sample slide thickness change the effective axial location of the object somewhat, so that these n propagation distances are slightly distinct from each other. Thus, MISHELF microscopy operates similarly to conventional single wavelength LHM with n slight axial movements of the digital sensor to capture n in-line Gabor holograms. 

After numerical propagation towards the object plane, the *n* focused images ($O_{1}^{\lambda_{1}},\;O_{1}^{\lambda_{2}},\;\ldots ,\;O_{1}^{\lambda_{n}}$) are utilized to generate a single merged image ($O_{1}$) by considering four steps. First, to avoid phase cancellation in the spectra mixing process, the global background phases of the *n* focused images must be set to have the same value. Second, a digital fast Fourier transform (FFT) of each focused image must be performed. Third, to generate a merged spectrum, the *n* spectra must be properly added with the application of a weighted mask so as not to generate low spatial frequency enhancement (spectral information provided by longer wavelengths is also supplied by shorter wavelengths). Fourth, an image of the sample ($\tilde{O_{1}}$) is recovered by inverse FFT computation of the merged spectrum, which is improved in terms of noise averaging and twin image reduction.

Then, the merged image is considered as the input of an iterative algorithm, which performs successive backward and forward digital propagations between both holograms and object planes, where certain conditions or constraints are imposed to the propagated complex amplitudes. In this iterative algorithm, the improved image $\tilde{O_{1}}$ is first divided into n complex amplitudes ($\tilde{O_{1}^{\lambda_{1}}},\;\tilde{O_{1}^{\lambda_{2}}},\;\ldots ,\;\tilde{O_{1}^{\lambda_{n}}}$), whose background phases are restored, and then propagated towards the hologram plane n times to get a new set of n in-line holograms, considering the n propagation distances previously obtained but with inverse sign. After that, the amplitudes of these new complex holograms ($B_{1}^{\lambda_{1}},\;B_{1}^{\lambda_{2}},\;\ldots ,\;B_{1}^{\lambda_{n}}$) are replaced by the square root of the hologram intensities originally captured ($A_{0}^{\lambda_{1}},\;\;A_{0}^{\lambda_{2}},\;\ldots ,\;A_{0}^{\lambda_{n}}$), while their phase distributions ($\varphi_{1}^{\lambda_{1}},\;\varphi_{1}^{\lambda_{2}},\;\ldots ,\;\varphi_{1}^{\lambda_{n}}$) are retained. Next to the generation of the new set of complex amplitudes ($\tilde{A_{1}^{\lambda_{1}}},\;\tilde{A_{1}^{\lambda_{2}}},\;\ldots ,\;\tilde{A_{1}^{\lambda_{n}}}$) at the hologram plane, we propagate them again to their focused planes to get the new focused images ($O_{2}^{\lambda_{1}},\;O_{2}^{\lambda_{2}},\;\ldots ,\;O_{2}^{\lambda_{n}}$). In the object plane, a merged spectrum is again generated to get a new single image $\tilde{O_{2}}$. Supplementary constraints can be also considered when a priori information of the object is known. Finally, this back-and-forth process with constraints imposed at the sample and hologram planes is repeated for each of the m cycles of the iterative algorithm to eventually achieve a final image $\tilde{O_{m}}$. Due to the high convergence of the algorithm, few iterations (typically two cycles) are required to provide the final image, which contains the information about the complex amplitude of the light diffracted by the sample and presents enhanced quality regarding contrast and noise. 

**Figure 3:**
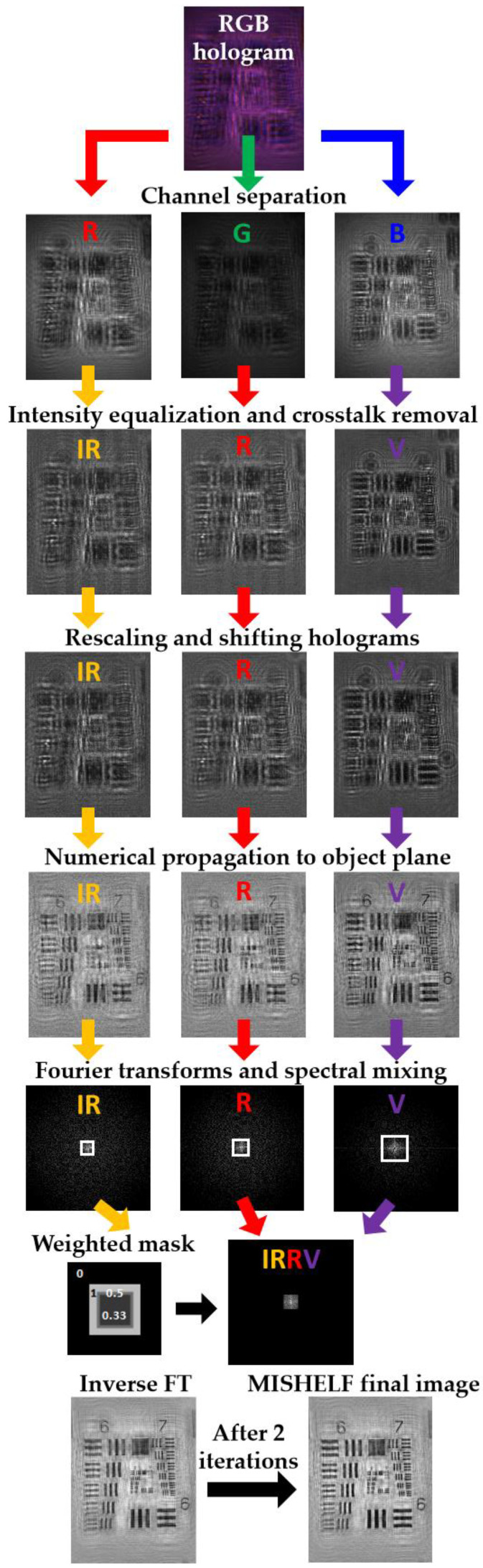
Example of the digital processing performed in MISHELF microscopy involving an USAF test target [22].

For a better understanding of the digital image processing, we present in Figure 3 a diagram of the process involving a positive USAF resolution target analyzed in [22], where the object was illuminated with infra-red, red and violet (IRRV) illumination wavelengths and a color RGB camera recorded the color holograms. The RGB in-line hologram is separated into its three detection channels and improved by intensity homogenization. Real-intensity holograms corresponding to the IRRV wavelength illuminations are then achieved after crosstalk removal and adjustment in relation to one another after rescaling and shifting. Three focused images at the sample plane are obtained after numerical backpropagation, which are Fourier transformed, and the merged spectrum is generated from the addition of such spectral information and considering a weighted mask. Such a merged spectrum is inverse Fourier transformed (FT) to achieve a better-quality image of the object plane, which is the input of the fast iterative algorithm. Finally, a final image with improved quality is provided by the MISHELF iterative algorithm after two iterations. It is important to mention that, although the iterative algorithm presents a rapid convergence, the implementation of several digital propagations per iteration reduces the computation speed, and the whole recovery process usually takes around a few seconds when it is implemented with a standard personal computer. 

## 3. Performance Analysis in MISHELF Microscopy

### 3.1. Performance Analysis

**Figure 4:**
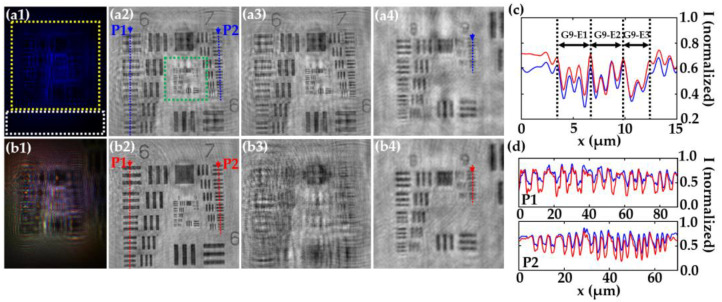
Performance analysis of MISHELF microscopy involving a positive USAF resolution test target [47]. Rows (**a**,**b**): results provided by B-LHM and MISHELF microscopy, respectively. (**a1**,**b1**) in-line color holograms, (**a2**,**b2**) amplitude reconstructions of the region of interest (yellow square in (**a1**)), (**a3**,**b3**) twin images, and (**a4**,**b4**) magnified central regions of images in (**a2**,**b2**) (green square in (**a2**)). (**c**) plots along blue and red arrows in (**a4**,**b4**) for spatial resolution comparison. (**d**) plots along blue and red arrows in (**a2**,**b2**) for contrast comparison. White rectangle area in (**a1**) is considered for coherence noise calculation.

In this section, we demonstrate the main characteristics of MISHELF microscopy regarding image reconstruction, spatial resolution, coherence noise reduction, contrast enhancement and twin image removal. For that purpose, we compare the performance of MISHELF microscopy employing three wavelengths (RGB illumination/detection channels) against the results obtained by conventional LHM with B illumination. The comparison is carried out involving a positive USAF resolution target, whose smallest elements can be regarded in good approximation as weak diffractive objects. Figure 4 shows the amplitude results of such a comparison when considering the MISHELF performance reported in [47]. The results for conventional B-LHM and MISHELF microscopy are included in first (a) and second (b) rows, respectively. First column (1) shows the recorded in-line holograms and the image reconstructions of the region of interest enclosed in the yellow rectangle in Figure 4(a1) are presented in the second one (2). Comparing both results, we can overall see a better image performance provided by MISHELF microscopy in comparison to LHM reconstruction.

As we previously mentioned, one of the most important characteristics of MISHELF microscopy is its capability to mitigate the presence of the twin image. To clearly demonstrate that, we include in Figure 4(a3,b3) the images obtained when propagating towards the twin image plane after application of LHM and MISHELF techniques, respectively. Looking at those images, one can notice the twin image mitigation provided by MISHELF microscopy in comparison to LHM, since there is almost not a focused USAF image at the twin image plane in contrast to LHM, where the same focused USAF image as in the image plane (see Figure 4(a2)) appears.

In addition, MISHELF microscopy provides high resolution imaging, whose spatial resolution is given by the lowest wavelength employed for illumination and considering Equation (4). To demonstrate that MISHELF microscopy does not incur any loss of resolution in comparison to conventional B-LHM, we compare a magnified area (green square in Figure 4(a2)) containing the finest elements of the USAF resolution target for the images provided by both methods (see Figure 4(a4,b4) for LHM and MISHELF reconstructions, respectively). In order to demonstrate that both images present the same resolution limit, we additionally include two plot profiles of the normalized intensity in Figure 4c along the dotted blue and red arrows included in Figure 4(a4,b4), respectively. From those profiles, we can see both LHM and MISHELF present the same resolution limit of 1.75 μm, since it corresponds to the resolution defined by the last element well-resolved, which is the element 2 of the group 9. 

Finally, to demonstrate the improvement of the image quality achieved by MISHELF microscopy in a quantitative way, we have analyzed the reduction in the coherence noise and increase in the contrast of the images. On one hand, we compare the background noise from standard deviation (STD) values at regions without an object of the normalized reconstructed images (marked with white rectangles in Figure 4(a1)) for both cases, achieving STD values of 0.038 and 0.025 for conventional B-LHM and MISHELF microscopy, respectively, which means a 34% reduction in the background noise. On the other hand, the contrast enhancement is analyzed from Figure 4d, where the plots along the blue and red arrows included in Figure 4(a2,b2) of the groups 6 (P1) and 7 (P2) are presented, respectively. The results provided by MISHELF show around 30% improvement in contrast to LHM.

### 3.2. Different Number of Illumination/Detection Channels

**Figure 5:**
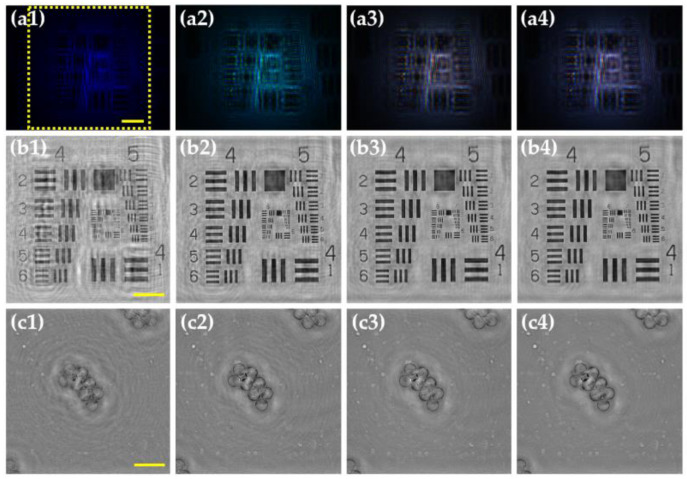
MISHELF microscopy implemented using different illumination/detection channels [49]. Rows (**a**,**b**): color holograms and amplitude reconstructions involving an USAF test target, respectively; row (**c**): phase images involving several 90 μm microspheres. (**a1**–**c1**): conventional LHM (V illumination); (**a2**–**c2**), (**a3**–**c3**) and (**a4**–**c4**): MISHELF microscopy implemented with two (V-G), three (V-G-R) and four (V-B-G-R) illumination wavelengths, respectively. Yellow scale bars in column (1) represent 100 μm.

MISHELF microscopy is not only limited to the use of three wavelengths for illumination, but it can be implemented utilizing as many illumination/detection channels as possible. For instance, MISHELF has been already implemented using two [48], three [22,49] and four [49] illumination/detection channels. Figure 5 includes a comparison of the results achieved by different number of channels involving an USAF test target and a cluster of air-immersed 90 μm polystyrene spheres [49]. Thus, results provided by conventional V-LHM are included in Figure 5(a1,b1,c1), whereas the results achieved with MISHELF microscopy when employing two (V-G), three (V-G-R) and four (V-B-G-R) illumination/detection channels are shown in Figure 5(a2–a4,b2–b4,c2–c4), respectively. In-line color holograms recorded by the whole sensitive area of the digital sensor are included in row (a) and the corresponding amplitude reconstructions of the central regions (see yellow square in Figure 5(a1)) are shown in row (b) involving the USAF test target, whereas phase results for the same region of the microsphere sample are included in row (c). As a general result, one can appreciate the global noise reduction and image contrast enhancement in both amplitude and phase distributions provided by MISHELF with the increment of the number of multiplexing channels.

## 4. MISHELF Microscopy: Microscope Prototypes

**Figure 6:**
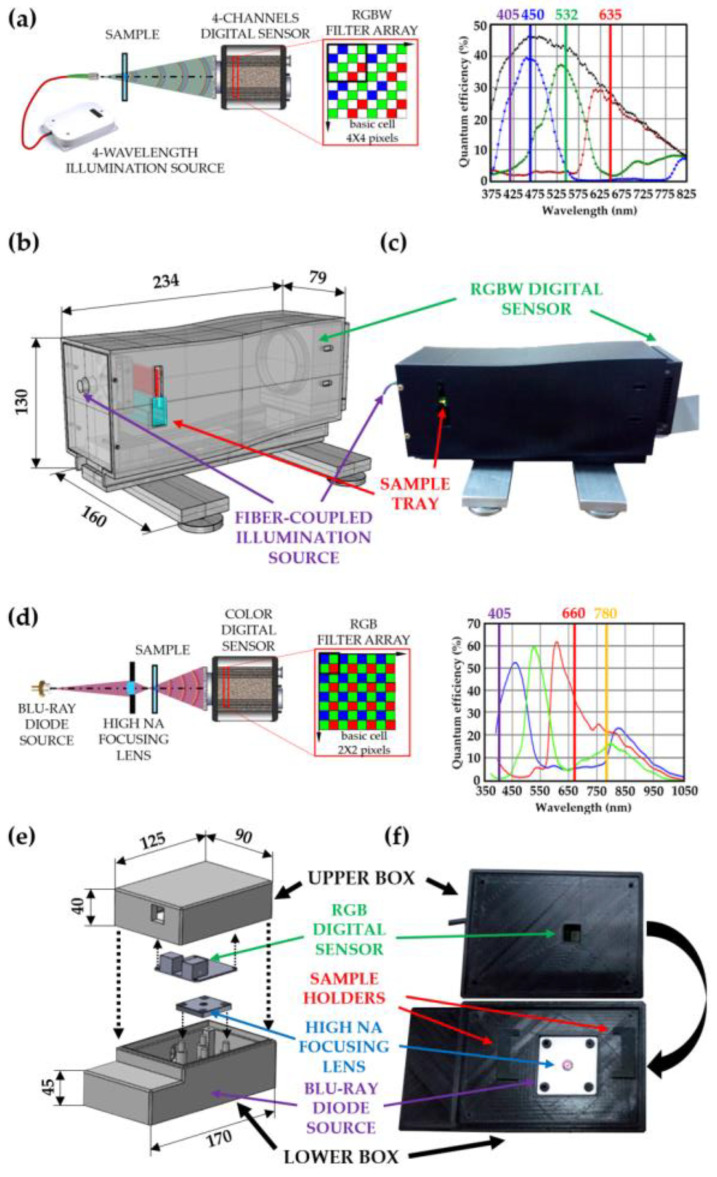
Microscope prototypes developed for MISHELF microscopy implementation. (**a**–**c**) High performance and (**d**–**f**) compact and cost-effective microscope prototypes, respectively. (**a**,**d**) Optical schemes of the microscopes including sensor pixel ordination, spectral sensitivity and wavelength line emissions; (**b**,**e**) perspective views of the prototypes design including units in mm; (**c**,**f**) pictures of fabricated microscopes.

Two different microscope prototypes have been developed to implement MISHELF microscopy in two different forms. First, a microscope prototype with expensive components (four RGBV fiber-coupled laser diodes and specific four-channel RGBW digital sensor) was designed to provide high performance (high contrast and reduced coherence-noise) and high quality image reconstructions employing four illumination/detection channels [49]. Second, a compact and cost-effective microscope prototype was fabricated for field portability [22], where a conventional RGB color camera is employed and the multiplexed coherent illumination is provided by three solid state lasers (infrared (IR), red (R) and (B) to read compact discs (CDs), digital versatile discs (DVDs), and Blu-ray discs (BDs), respectively) included in a small and inexpensive diode can contained in a Blu-ray optical unit. Figure 6 presents an overview of the main features of the high performance (see Figure 6a–c) and cost-effective (see Figure 6d–f) microscope prototypes reported in [22,49], respectively. Figure 6a,d shows a scheme of the optical layouts including a representation of the pixel ordination of the digital sensors. We additionally include in Figure 6a,d a plot with the spectral sensitivities of the different filter arrays of the digital sensors together with the wavelength emission lines provided by the light sources. Furthermore, Figure 6b,e shows a perspective view of the design of the prototypes including the external dimensions in mm. Both microscope prototypes were designed using a commercially available CAD software platform. Finally, Figure 6c,f includes the pictures of the manufactured microscope prototypes, where the main elements are indicated. Those prototypes were built with a 3D printer employing fused deposition material containing ABS for the majority of the parts and with a mechanization process for the other parts.

## 5. Biomedical Applications of MISHELF Microscopy to Sperm Motility Analysis

So far, MISHELF microscopy has been mostly applied in biomedicine to the analysis of sperm motility. The diagnosis of male fertility is based on semen analysis, where sperm motility plays a crucial role in its prediction [92]. Conventional methods for sperm motility analysis are mostly based on incoherent optical microscopy techniques (normally using 10 X and 20 X microscope lenses), which only provide information about the cells contained within a limited DOF of typically few microns (therefore losing the information of those cells out of focus). That means that routine motility analyses are performed using shallow counting chambers (typically with depths of 10 or 20 μm) [33]. However, the use of shallow counting chambers affects to the intrinsic sperm motion, since they impede the intrinsic helical movement of the sperm tail (human spermatozoon length is approximately 50 μm), which results in an artificial motility pattern. 

**Figure 7:**
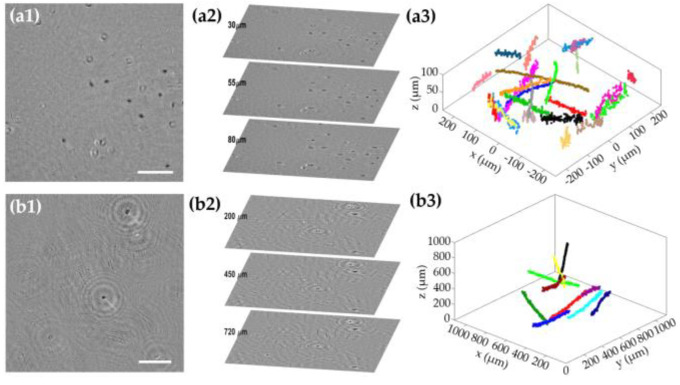
Sperm motility analysis performed with MISHELF microscopy involving different types of sperm cells. Rows (**a**,**b**) human and starry skate fish spermatozoa, respectively. (**a1**,**b1**) reconstructed amplitude images in a sample plane; (**a2**,**b2**) perspective views of three different planes showing sperm cells in focus; (**a3**,**b3**) 3D trajectories of some spermatozoa. Scale bars in (**a1**,**b1**) represent 100 and 200 μm, respectively.

As other LHM techniques, MISHELF microscopy is a holographic technique that can provide, from a single holographic recording, the amplitude and phase information at different axial planes beyond the DOF, thus enabling the 3D analysis of motility patterns utilizing counting chambers with depths of >50−100 μm, thus being a perfect tool for sperm motility assessment. In addition, MISHELF microscopy also provides high-contrast imaging with reduced noise and twin image mitigation, which allows a better identification and analysis of spermatozoa features. Because of that, MISHELF microscopy has been employed for the analysis of many different types of sperm cells, mainly covering both mammalian and fish samples. Figure 7 includes representative frames of some of the results achieved from the application of MISHELF microscopy in the case of human (see row (1) and Videos S1–S3) and starry skate fish (see row (2) and Videos S4–S6) sperm cell samples, swimming within counting chambers with depths of 100 μm and 1 mm, respectively. Figure 7(a1,b1) and Videos S1 and S4 show amplitude images reconstructed into a sample plane containing several focused sperm cells. Due to the counting chambers having a depth greater than the DOF provided by the microscope, sperm cells located in other axial planes appear defocused. However, those sperm cells can be brought into focus by numerical propagation, so that different sperm cells at different axial locations can be represented at the same time, as shown in Figure 7(a2,b2) and Videos S2 and S5, where the images of three different sample planes are included in a perspective representation to clearly identify the amount of spermatozoa contained in each plane. Finally, different focusing criteria [93,94,95,96] can be applied to the images to obtain the XYZ positions of the sperm cells for each time lapse, and to eventually compute the trajectories in 3D space taken by the spermatozoa during the investigation time (some representative trajectories are included along Figure 7(a3,b3) and Videos S3 and S6). 

**Figure 8:**
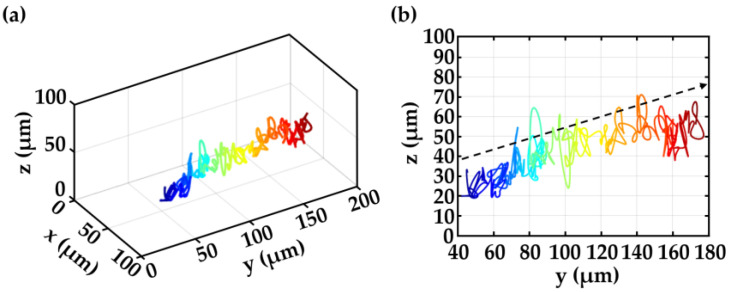
Example of a 3D trajectory of a boar sperm cell provided by MISHELF microscopy [33]: (**a**) perspective and (**b**) lateral views, respectively. Black arrow in (**b**) highlights the axial movement of the spermatozoon.

In another study, MISHELF microscopy has been employed for sperm motility analysis to evaluate the impact of the depth of the counting chambers on the kinematic parameters of sperm cells [33]. The determination of the distinct motility patterns investigated in counting chambers with different depths (10, 20 and 100 μm) has been performed for the case of boar sperm cells [33]. In that study, MISHELF microscopy has been combined with a specific commercial software for sperm motility analysis (CASA-Mot system [97]) to provide information about multiple kinematic parameters. The experimental results showed a good agreement between results provided by MISHELF microscopy compared to the results provided by well-established routine techniques (ISAS^®^v1 CASA-Mot system) for the case of 20 μm counting chambers, meaning that the technique is capable of performing routine analysis of boar semen equivalently to conventional optical CASA-Mot systems. In addition, the experimental results also showed that important motility features such as curvilinear velocity, straight line velocity, wobble and beat cross frequency were statistically higher in the case of 100 μm chambers in comparison to the 10 μm and 20 μm chambers. That means that the increase in counting chamber depth has an important impact on the kinematic parameters of the spermatozoa, becoming higher when increasing the chamber depth, revealing a more natural sperm motion within 100 μm counting chambers that is not affected by the chamber surfaces. This fact highlights the significance of using imaging techniques such as MISHELF microscopy with extended DOF for natural sperm motility analysis. Finally, MISHELF microscopy could additionally provide a set of unknown 3D kinematic parameters, which could enhance the insights in sperm motility behavior in diverse natural environments [30,31,32]. Just as a final example of the significance of employing technologies for 3D sperm motility analysis, Figure 8a,b shows a perspective and a lateral view of the 3D trajectory of a boar sperm cell, respectively, provided by MISHELF microscopy, moving in a straight line but having an axial out-of-focus displacement (as indicated by the black arrow in Figure 8b), which could not be traced/analyzed by conventional methods with limited DOF. 

## 6. Conclusions and Outlook

In summary, MISHELF microscopy is a label-free imaging technique that allows single-shot 3D analysis of fast dynamic events. MISHELF microscopy is an LHM technique which employs a wavelength multiplexing approach and a fast-convergence iterative algorithm to improve the quality of holographic (amplitude and phase) image reconstructions. In MISHELF microscopy, the quality enhancement of the image is caused by the mitigation of the twin image, the reduction of the coherence noise and the enhancement of the contrast. For that, MISHELF illuminates a sample with several illumination wavelengths at the same time, and a color digital sensor records the resulting color in-line Gabor holograms at video rate speed. Then, those color multiplexed holograms are the inputs for the iterative algorithm, which requires only a few cycles for achieving high-quality imaging. MISHELF microscopy has already been implemented and experimentally validated in optical table arrangements [47,48] as well as in different types of microscope prototypes [22,49].

The MISHELF technique is versatile in the number of illumination/detection channels as well as in the tuning between the emission wavelengths of the light sources and the spectral sensitivity of the digital sensor. In principle, MISHELF has no theoretical limitation on the number of wavelengths to be used. However, in practice, there exist nowadays hardware restrictions due to the limitations of the light sources, or more strongly restrictive, limitations in the number of detection channels provided by current digital cameras. Furthermore, MISHELF does not introduce any spatial resolution reduction in the optical system in comparison to other LHMs. In addition, because the complex amplitude is recovered from a single snapshot, the frame rate of the digital camera is the only limiting factor for imaging rapid events, therefore allowing video-rate analysis of dynamic biological samples. 

Regarding the drawbacks of MISHELF microscopy, the use of a wavelength multiplexing approach introduces an inherent limitation with respect to the analysis of color selective samples. Those samples present wavelength-dependent differences in absorption and dispersion, whose color information is sacrificed to achieve high-quality holographic imaging. In addition, those differences could result in different hologram intensities, which can in turn lead to the lack of information for those high absorption regions. That problem could be mitigated, for instance, by employing multi-wavelength light sources including more wavelength illuminations than used and by selecting the most appropriated wavelengths for the specific application, or by controlling the light intensity coming from the different wavelength illuminations. Another drawback of MISHELF microscopy is the restriction in the dynamic range presented when a single detection channel of the digital camera simultaneously detects several illumination wavelengths, being more restrictive when the number of illuminations detected by the channel is higher. Concerning the drawbacks of the MISHELF algorithm, the consumption time of a few seconds per recovered image could be disadvantageous in applications where high-speed analysis is required. Furthermore, the image recovery procedure of MISHELF microscopy could be relatively more laborious and complex to be implemented by non-expert scientists in the field than other LHM algorithms. 

To conclude, MISHELF microscopy is a robust and versatile technique with great possibilities in biomedicine. Highly dynamic biological samples, such as sperm cells, have emphasized the importance of MISHELF microscopy for diverse biomedical applications. Following the research performed in [33], MISHELF microscopy can be further applied for sperm analysis. Sperm assessment is nowadays a highly attractive and paramount research field for the evaluation of male fertility and its perfection in animal husbandry [30,31,98,99,100]. Understanding the biology of sperm could one day help treat infertile couples and improve human in vitro fertilization and animal reproduction [101,102,103]. Previous works reported several infrequent swimming patterns [30,102,103,104]. In that sense, MISHELF microscopy has been shown to be capable of providing high-resolution sperm imaging and 3D trajectory of sperm cells with compact and cost-effective microscope prototypes, being a microscopy technique suited for this kind of applications. In addition, other future biomedical applications could be developed, for instance, for the combination of MISHELF microscopy with microfluidics to allow imaging flow cytometry [105,106] or to combine the technique with deep learning-assisted techniques [86,107,108] for the diagnosis of cancer and diseases [23,24,25,78,109]. Finally, the miniaturization of the MISHELF technique through the development of compact and cost-effective microscopes makes MISHELF microscopy suitable for a field setting or telemedicine applications in resource-limited environments [2]. 

## Data Availability

Not applicable.

## Supplementary Materials

The following supporting information can be downloaded at: https://www.mdpi.com/article/10.3390/s23031472/s1, Video S1: amplitude reconstructions in a sample plane of a human sperm sample; Video S2: perspective views of amplitude reconstructions of three different planes of a human sperm sample; Video S3: 3D trajectories of human spermatozoa; Video S4: amplitude reconstructions in a sample plane of a starry skate fish sperm sample; Videos S5: perspective views of amplitude reconstructions of three different planes of a starry skate fish sperm sample; Video S6: 3D trajectories of starry skate fish spermatozoa.
